# 

**Published:** 1864-04

**Authors:** 


### CONTENTS.

### ORIGINAL CONTRIBUTIONS.

ART. XIII. VALEDICTORY ADDRESS TO THE GRADUATING CLASS IN CHICAGO MEDICAL COLLEGE, MEDICAL DEPARTMENT LIND UNIVERSITY, at the Fifth Annual Commencement, March 3, 1864. By HENRY WING, M.D., Prof. of General Pathology, &c., ..................... 193
ART. XIV. IMPROVED METHODS OF COUNTER-EXTENSION IN TREATMENT OF FRACTURES OF THE INFERIOR EXTREMITIES. By E. ANDREWS, M.D., Professor of Surgery in Chicago Medical College,......... 207
ART. XV. ABSCESS OF THE GALL-BLADDER—RUPTURE AND EVACUATION INTO THE PERITONEAL CAVITY—DEATH—AUTOPSY. By HENRY M. LYMAN, of Chicago, ........................................ 210
ART. XVI. RUPTURE OF UTERUS. By E. HOLDERNESS, M.D., of Towanda, McLean Co., Ill.,........................................ 216
ART. XVII. CASES OF BAD VACCINATION IN THE ARMY. By GEO. O. SMITH, M.D., Asst.-Surgeon 53d Regiment Ill. Vols., ....................... 218
ART. XVIII. NOTES OF A CASE OF ERYSIPELAS. By D. W. YOUNG, M.D., of Aurora, Ill. (Read before the Aurora City Medical Association,)........................................ 220

### SELECTIONS.

On the Treatment of Rheumatic Fever. By Dr. J. Birbeck Nevins, Lecturer on Materia Medica, Royal Infirmary School of Medicine, Liverpool, ........................................ 226
On Gout, Rheumatism, Rheumatic Gout, and Sciatica. By Henry W. Fuller, Physician to St. George's Hospital, ........................ 230
New Disease,........................................ 238
On Iodine as a Deodoriser and Disinfectant. By Dr. B. W. Richardson, M.A., London,........................................ 241
Mercury as a Remedy. Letter from Thomas Pope, Esq.,..................... 242
University of London,........................................ 243

### BOOK NOTICES.

Transactions of the American Medical Association, Vol. XIV. 1864,..... 244
A Treatise on Human Physiology. By John C. Dalton, Jr., M.D.,.......... 244
Lectures on Orthopædic Surgery. By Louis Bauer, M.D.,.................. 245
A Case of Neuroma on the Optic Nerve. By John A. Lidell, M.D.,........ 245
Annual Report of the Commissioners, Superintendent, and Treasurer of the Indiana Hospital for the Insane; for the year 1863,................ 245
Twenty-first Annual Report of the Managers of the New York State Lunatic Asylum, for the year 1863,........................................ 245

### EDITORIAL.

Death of Dr. O. L. Leonard, ........................................ 246
Chicago Medical Society, ........................................ 246
Annual Meeting of the Illinois State Medical Society,........................ 247
The People's Dental Journal,........................................ 247
Acknowledgment,........................................ 247
Transactions of the American Medical Association, Vol. XIV., .............. 248
The Relations of Height and Weight in the Human Body,.................. 249
Use and Abuse of Stimulants in Fever—Occasional Antiphlogistic Treatment,........................................ 249
Treatment of Dysentery by Large Doses of Ipecacuanha,.................. 250
Extraordinary Longevity, ........................................ 251
M. Velpeau at the Academy of Sciences of Paris, ........................ 251
The British Pharmacopœia,........................................ 252
Infantile Mortality in New York City, ........................................ 252
Action for Alleged Illegal Detention in an Asylum, ........................ 253

